# Clinical implications of serum *N‐*glycan profiling as a diagnostic and prognostic biomarker in germ‐cell tumors

**DOI:** 10.1002/cam4.1035

**Published:** 2017-03-20

**Authors:** Takuma Narita, Shingo Hatakeyama, Tohru Yoneyama, Shintaro Narita, Shinichi Yamashita, Koji Mitsuzuka, Toshihiko Sakurai, Sadafumi Kawamura, Tatsuo Tochigi, Ippei Takahashi, Shigeyuki Nakaji, Yuki Tobisawa, Hayato Yamamoto, Takuya Koie, Norihiko Tsuchiya, Tomonori Habuchi, Yoichi Arai, Chikara Ohyama

**Affiliations:** ^1^ Department of UrologyHirosaki University Graduate School of MedicineHirosakiJapan; ^2^ Department of Advanced Transplant and Regenerative MedicineHirosaki University Graduate School of MedicineHirosakiJapan; ^3^ Department of UrologyAkita University Graduate School of MedicineAkitaJapan; ^4^ Department of UrologyTohoku University Graduate School of MedicineSendaiJapan; ^5^ Department of UrologyYamagata University Graduate School of MedicineYamagataJapan; ^6^ Department of UrologyMiyagi Cancer CenterNatoriJapan; ^7^ Department of Social MedicineHirosaki University School of MedicineHirosakiJapan

**Keywords:** Biomarker, germ‐cell tumors, glycoblotting, mass spectrometry, serum *N‐*glycan

## Abstract

Serum biomarker monitoring is essential for management of germ‐cell tumors (GCT). However, not all GCT are positive for conventional tumor markers. We examined whether serum *N*‐glycan‐based biomarkers can be applied for detection and prognosis in patients with GCT. We performed a comprehensive *N*‐glycan structural analysis of sera from 54 untreated GCT patients and 103 age‐adjusted healthy volunteers using glycoblotting methods and mass spectrometry. Candidate *N‐*glycans were selected from those with the highest association; cutoff concentration values were established, and an *N‐*glycan score was created based on the number of positive *N‐*glycans present. The validity of this score for diagnosis and prognosis was analyzed using a receiver operating characteristic (ROC) curve. We identified five candidate *N‐*glycans significantly associated with GCT patients. The accuracy of the *N‐*glycan score for GCT was significant with an area‐under‐the‐curve (AUC) value of 0.87. Diagnostically, the *N‐*glycan score detected 10 of 12 (83%) patients with negative conventional tumor markers. Prognostically, the *N‐*glycan score comprised four candidate *N*‐glycans. The predictive value of the prognostic *N*‐glycan score was significant, with an AUC value of 0.89. A high value prognostic *N*‐glycan score was significantly associated with poor prognosis. Finally, to identify a potential carrier protein, immunoglobulin (Ig) fractions of sera were subjected to *N*‐glycan analysis and compared to whole sera. Candidate *N*‐glycans in Ig‐fractions were significantly decreased; therefore, the carrier protein for candidate *N*‐glycans is likely not an immunoglobulin. In summary, our newly developed *N*‐glycan score seems to be a practical diagnostic and prognostic method for GCT.

## Introduction

Germ‐cell tumors (GCT) remain the most common solid malignancy in young men 1. Serum tumor markers are important for diagnosis and risk stratification as prognostic predictors. However, conventional tumor markers, including human chorionic gonadotropin (HCG), alpha‐fetoprotein (AFP), and lactate dehydrogenase (LDH), are elevated in <60% of patients 2. Up to 30% of seminomas present with an elevated HCG level. AFP and HCG are increased in 50–70% and in 40–60% of patients with non‐seminomatous GCT (NSGCT), respectively 3. In addition, because cisplatin‐based chemotherapy is highly effective for treating advanced cases, the evaluation of residual retroperitoneal lesions using conventional tumor markers is challenging after conversion to being marker negative. Therefore, novel and sensitive tumor biomarkers are needed.

Glycosylation is a very common posttranslational modification, and it is estimated to happen >70% in case of proteins 4. Glycans are known to have crucial roles in molecular recognition and cell‐to‐cell interactions, and disorders of the glycan system are related to many diseases, especially cancer 5. Although several glycomics studies have been reported for GCT, these findings were from cell lines or tumor specimens 6, 7. Recently, our group established a chemoselective glycan‐enrichment technology, called glycoblotting, to purify oligosaccharides from a crude glycoprotein mixture 8, and revealed that serum *N*‐glycomics has promise to screen for a diagnostic and prognostic marker for both renal‐cell carcinoma and castration‐resistant prostate cancer 9, 10. It also has promise as a prognostic tool in patients undergoing hemodialysis 11. However, glycan‐based serum biomarkers for GCT have not yet been established. In this study, we examined serum *N*‐glycans in GCT patients using the glycoblotting method and evaluated whether serum *N*‐glycan profiling can be applied for detection and prognosis in patients with GCT.

## Materials and Methods

This study was performed in accordance with the ethical standards of the Declaration of Helsinki and approved by the institutional ethics committee of Hirosaki University Graduate School of Medicine (authorization number, 2015‐144). Written or verbal informed consent was obtained from all serum donors.

From April 2012 to September 2016, a total of 54 patients with GCT who were scheduled to undergo orchiectomy or systemic chemotherapy were recruited in a total of five institutions (Tohoku University Hospital, Hirosaki University Hospital, Akita University Hospital, Yamagata University Hospital, and Miyagi Cancer Center). Serum samples were obtained from 42 patients (GCT group) before treatment and stored at −80°C. Control samples obtained from our serum bank comprised 358 volunteers and were stored at −80°C. The patient's characteristics at diagnosis, including age, clinical stage, and conventional tumor markers (HCG, AFP, LDH) were recorded before starting treatment. All tumors were staged according to the 2009 tumor–node–metastasis classification 12 and risk stratification was evaluated according to the International Germ‐cell Consensus Classification (IGCCC) 13. Control samples were obtained from community‐dwelling volunteers in Akita University Hospital, Hirosaki University Hospital, and the health maintenance program in the Iwaki Health Promotion Project. Because of the retrospective nature of this study, we included 103 age‐adjusted control subjects (HLT group) in this study. We compared *N‐*glycan concentrations (*μ*M) between the HLT and the GCT groups to select candidate *N*‐glycan moieties for detecting the presence of GCT by area‐under‐the‐curve (AUC) values. Optimal cutoff values were determined by receiver operating characteristic (ROC) curves. Candidate *N*‐glycans were defined as a positive when the concentration was higher than the cutoff value. Diagnostic and prognostic N‐glycan scores were created by adding the number of candidate *N‐*glycans over the concentration cutoff value. The accuracy of the *N‐*glycan scores was evaluated by ROC analysis. The glycan symbol in this study followed the instruction of symbol nomenclature for glycan representation.

### Glycoblotting method and mass spectrometry

Serum *N*‐glycan analysis was performed as described previously using SweetBlot^TM^ (System Instruments, Hachijo, Japan) 8. Briefly, 10 *μ*L of serum samples containing 40 pmol of the internal standard disialo‐galactosylated bi‐antennary *N*‐glycan, which has amidated sialic acids (A2 amide glycans) (Table S2), were reduced and alkylated using DTT and iodoacetamide (Wako Pure Chemical Industries, Osaka, Japan), respectively. The resulting mixture was then trypsinized and heat inactivated. After cooling down to room temperature, peptide *N*‐glycanase F (New England BioLabs, Ipswich, MA) was added to the mixture to release total serum *N‐*glycans. After incubating for 360 min at 37°C, 20 *μ*L of the resulting mixture, equivalent to 2.5 *μ*L of serum, was mixed with 500 *μ*L of BlotGlyco H beads (Sumitomo Bakelite, Co., Tokyo, Japan) to capture glycans via stable hydrazone bonds on MultiScreen Solvinert^®^ filter plate (MerkMillipore, Billerica, MA). Then, acetyl capping of unreacted hydrazide functional groups on the beads and methyl esterification of sialic acid carboxyl groups, which exist at the terminal ends of the captured glycans, were performed sequentially; serial washes were then performed before each step, as described previously 9, 10, 11, 14. The captured *N‐*glycans were labeled with BOA (Sigma–Aldrich, St. Louis, MO) by transiminization and were eluted in 150 *μ*L of water. The BOA‐labeled glycans were detected using matrix‐assisted laser desorption/ionization‐time of flight (MALDI‐TOF) mass spectrometry (Ultraflex 3 TOF/TOF mass spectrometer, Bruker Daltonics, Bremen, Germany). Compositions and structures of glycans were predicted using GlycoMod Tool (http://br.expasy.org/tools/glcomod). The quantitative reproducibility test of Sweetblot was performed as described previously 14.

### Purification of Immunoglobulin (Ig) fraction from serum

To determine the *N‐*glycan profile of serum immunoglobulin (Ig), Ig purification was performed using a Zeba^TM^ Spin Desalting Plate and Melon^TM^ Gel Spin Purification Kit (Thermo Fisher Scientific, Waltham, MA) according to the manufacturer's instruction. Briefly, the Zeba desalting plate was placed on top of wash plate and centrifuged at 1000*g* for 2 min to remove the storage buffer. Then, 250 *μ*L of PBS was added on top of the Zeba desalting resin, centrifuged at 1000*g* for 2 min, and the flow‐through discarded. This step was repeated an additional three times. Then, the desalting plate was stacked on top of the sample collection plate. Serum samples (100 *μ*L) were applied to the center of the Zeba desalting resin and centrifuged at 1000*g* for 2 min. Flow‐through was collected as buffer‐exchanged serums and was stored at −80°C until use. The Melon gel spin plate was placed on top of the wash plate and centrifuged at 1000*g* for 2 min to remove the storage buffer. Then, 300 *μ*L of purification buffer was added on top of the Melon gel resin and centrifuged at 1000*g* for 2 min, and the flow‐through discarded. This step was repeated once. Then, the Melon gel plate was stacked on top of the sample collection plate. Buffer‐exchanged serum samples (100 *μ*L) were applied to the center of the Melon gel resin and centrifuged at 1000*g* for 2 min. Flow‐through was collected as a purified Ig‐fraction. An Ig‐fraction of 10 *μ*L was subjected to our glycoblotting method and mass spectrometry.

### Statistical analysis

Statistical analyses of clinical data were performed using SPSS v. 22.0 (IBM Corporation, Armonk, NY) and GraphPad Prism v. 5.03 (GraphPad Software, San Diego, CA). Categorical variables were reported as percentages and compared using Fisher's exact test. Quantitative data were expressed as medians with quartiles 1 and 3 (Q1, Q3). Differences between the groups were statistically compared using a Student's *t*‐test for data with normal distribution or a Mann–Whitney *U*‐test for data exhibiting a non‐normal distribution. The optimal cutoff values of intensity of *N‐*glycans for UC detection were calculated using the following formula: (1−sensitivity)^2^ + (1−specificity)^2^ with the ROC curve. Independent factors influencing on UC diagnosis were identified by multivariate analyses using a logistic regression model. Odds ratios with 95% confidence intervals (95% CI) were calculated after concurrently adjusting for potential confounders.

## Results

The backgrounds of the HLT and GCT groups are shown in Table 1. There were no statistically significant differences in age between the groups. Half of the patients (*n* = 22, 52%) had NSGCT in pathological findings, and a majority of patients were Stage I (38%) or III (36%) in the GCT group. About metastatic disease, 64% of patients were in the good prognosis group. Twelve patients (29%) were negative for any conventional tumor markers.

**Table 1:** Background of patients

	HLT	GCT	*P* value
*N*	103	42	
Age	37.7 ± 16.3	36.8 ± 11.5	0.789
Seminoma		20 (48%)	
Non‐seminoma		22 (52%)	
Stage			
I		16 (38%)	
II		7 (17%)	
III		15 (36%)	
Extragonadal		4 (9.5%)	
IGCCC			
Good		16 (64%)	
Intermediate		6 (24%)	
Poor		4 (16%)	
Relapse		5 (12%)	
Deceased		3 (7%)	

### Selection of diagnostic *N‐*glycans

**Figure 1:**
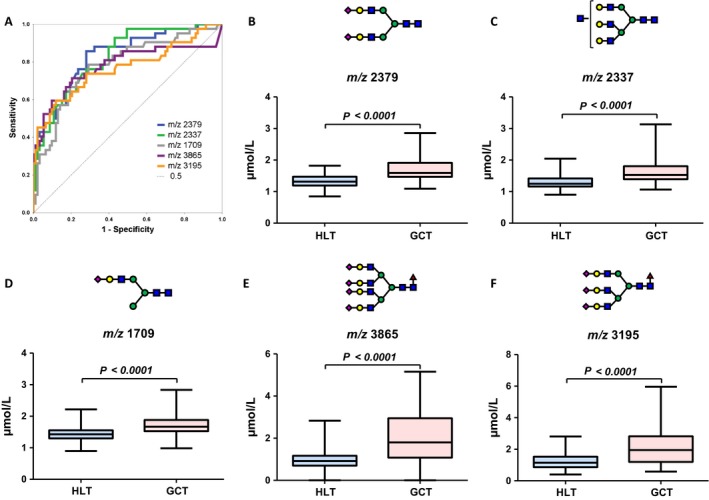
Selection for diagnostic *N‐*glycans. (A) Five *N‐*glycans (*m/z* 2379, 2337, 1709, 3865, 3195) with AUC >0.75 by receiver operating characteristic curves were selected.(B, C, D, E, and F) Concentrations of these diagnostic *N‐*glycan were significantly increased in the GCT group.

**Figure 2:**
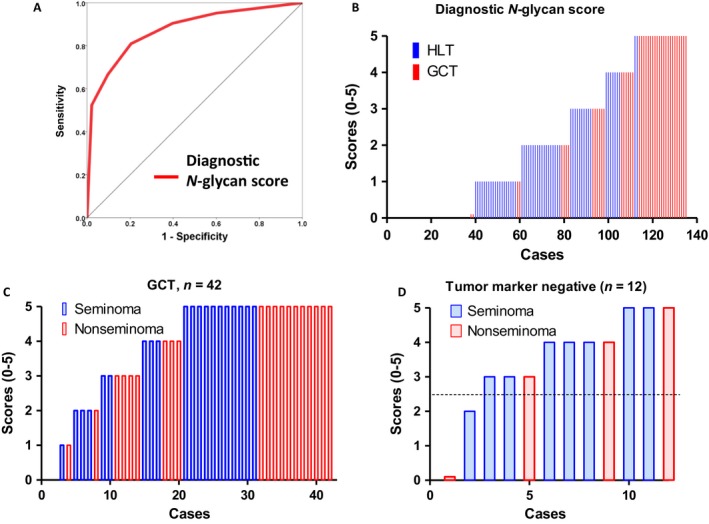
The predictive value of the diagnostic *N‐*glycan score for GCT. (A) The diagnostic *N‐*glycan score was created by five candidate *N‐*glycans (*m/z* 2379, 2337, 1709, 3865, 3195). The diagnostic value of *N‐*glycan score was significant with the AUC value of 0.87 (95% CI: 0.81–0.94, *P* < 0.001). (B) GCT patients had higher *N‐*glycan scores than the HLT patients. (C) There was no significant difference in diagnostic *N‐*glycan score between seminoma and non‐seminomatous GCT (*P* = 0.561). (D) The diagnostic *N‐*glycan score detects 10 of 12 (83%) patients with negative conventional tumor markers.

Serum *N‐*glycomics performed using the glycoblotting method and mass spectrometry identified 70 types of BOA‐labeled *N‐*glycans in all serum samples. After quantitative reproducibility tests, statistical analysis indicated that 36 types of *N‐*glycans (Table S1) had quantitative reproducibility among all samples. Of those, we selected five *N‐*glycans (*m/z* 2379, 2337, 1709, 3865, 3195) with AUC that were >0.75 by ROC curves (Fig. 1A). Unselected *N‐*glycans with AUC under 0.70 are shown in Supplemental Figure 1 (Fig. S1). *N‐*glycan levels of complex‐type glycans were significantly increased in the GCT group (Figs. 1B, C, D, E, and F). Table 2 shows *N‐*glycans with AUC values >0.70, and the optimal cutoff values for the five selected *N‐*glycans. The sum of the positive number of candidate *N‐*glycans for GCT detection, which was defined as a diagnostic *N‐*glycan score, was significantly higher in the GCT group than in the HLT group (5.0 vs. 1.0, *P *<* *0.001, respectively). The predictive value of diagnostic *N‐*glycan score for GCT was significant with the AUC value of 0.87 (95% CI: 0.81–0.94, *P *<* *0.001) (Fig. 2A). The optimal cutoff value was identified as 2.5 points with 81% sensitivity and 80% specificity (Tables 2 3). Bar plotting of the *N‐*glycan score in each subject showed that the GCT patients had higher *N‐*glycan scores (red bars) than the HLT patients (blue bars) (Fig. 2B). There was no significant difference in diagnostic *N‐*glycan score between seminoma and NSGCT (*P *=* *0.561) (Fig. 2C). A diagnostic *N‐*glycan score detected 10 of 12 (83%) patients with negative tumor markers (Fig. 2D).

**Table 2:** Significant *N*‐glycans and their optimal cutoff values associated with GCT detection, IGCCC risk classification (intermediate or poor risk), and progression

Diagnosis	AUC	P	95% CI	Cut off value
*m/z* 2379	0.84	<0.001	0.76–0.91	1.4280
*m/z* 2337	0.83	<0.001	0.75–0.90	1.3975
*m/z* 1709	0.78	<0.001	0.69–0.87	1.5255
*m/z* 3865	0.78	<0.001	0.68–0.88	1.1945
*m/z* 3195	0.77	<0.001	0.67–0.86	1.4375
*m/z* 2890	0.74	<0.001	0.65–0.84	
*m/z* 3719	0.73	<0.001	0.64–0.82	
*m/z* 3414	0.72	<0.001	0.63–0.81	
*m/z* 3560	0.72	<0.001	0.60–0.83	
*m/z* 2011	0.71	<0.001	0.62–0.80	
*m/z* 3049	0.70	<0.001	0.61–0.80	
Advanced disease				
* m/z* 3560	0.75	0.020	0.56–0.93	
* m/z* 3109	0.74	0.026	0.56–0.92	
* m/z* 2525	0.73	0.031	0.58–0.88	
* m/z* 3865	0.72	0.037	0.52–0.93	
* m/z* 2890	0.71	0.045	0.53–0.90	
* m/z* 3195	0.70	0.055	0.51–0.89	
Progression				
* m/z* 2890	0.81	0.028	0.68–0.93	2.1255
* m/z* 3195	0.77	0.050	0.60–0.95	2.8215
* m/z* 3865	0.74	0.087	0.49–0.99	2.6665
* m/z* 3560	0.76	0.065	0.56–0.96	2.8720
*N*‐glycan score				
Diagnostic	0.87	<0.001	0.81–0.94	2.5
Prognostic	0.89	0.005	0.78–1.00	3.5

AUC, area‐under‐the‐curve; 95% CI, 95% confidence interval.

**Table 3:** Effect on *N*‐glycan score on sensitivity, specificity, positive predictive value (PPV), and negative predictive value (NPV) at multivariable model cutoff

Cutoff value	% Sensitivity	% Specificity	% PPV	% NPV
0.5	95	40	42	95
1.5	91	60	51	93
2.5	81	80	64	90
3.5	67	90	76	86
4.5	52	98	92	82

### Selection of prognostic *N‐*glycans

**Figure 3:**
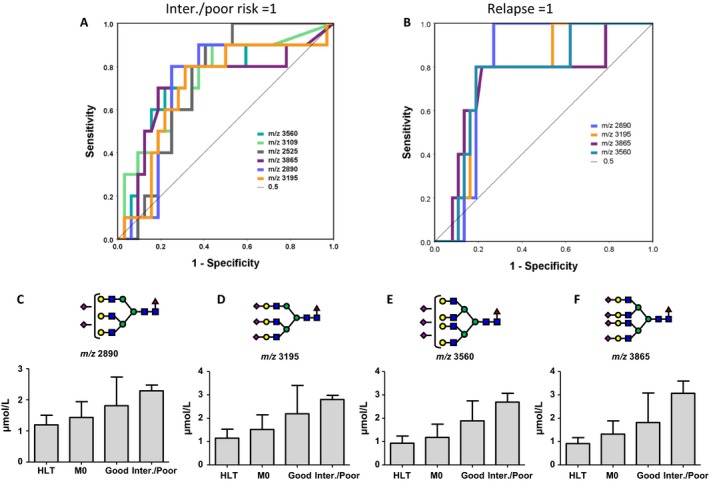
Selection for *N‐*glycans related with advanced disease status. (A) Six *N‐*glycans (*m/z* 3560, 3109, 2525, 3865, 2890, and 3195) were selected as advanced disease‐related *N‐*glycans. (B) Four *N‐*glycans (*m/z* 2890, 3195, 3560, and 3865) were selected as relapse‐related *N‐*glycans. (C, D, E, and F) Increasing prognostic *N‐*glycan levels were associated with advanced disease status.

We performed ROC analysis to select prognosis‐related *N‐*glycans. *N‐*glycans of *m/z* 3560, 3109, 2525, 3865, 2890, and 3195 were selected as advanced disease (intermediate or poor prognosis‐related) *N‐*glycans (Fig. 3A). *N‐*glycans of *m/z* 2890, 3195, 3560, and 3865 were selected as relapse‐related *N‐*glycans (Fig. 3B). Candidate *N‐*glycans with optimal cutoff values are shown in Table 2. A prognostic *N‐*glycan score was created utilizing four candidate *N‐*glycans (*m/z* 2890, 3195, 3560, and 3865) that were significantly associated with both advanced disease and tumor progression. Increased prognostic *N‐*glycan scores were associated with advanced disease status (Fig. 3C, D, E, and F).

**Figure 4:**
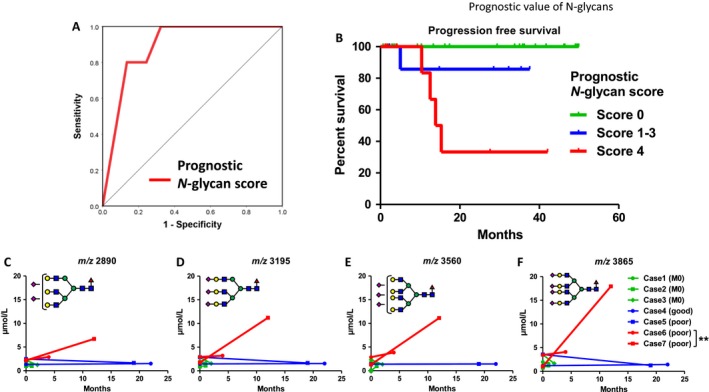
The predictive value of prognostic *N‐*glycan scores for Germ‐cell tumors. (A) The predictive value of the prognostic *N‐*glycan score was significant with an AUC value of 0.89. (B) A high prognostic *N‐*glycan score is significantly associated with poor prognosis (*P *=* *0.0012). (C, D, E, and F) Longitudinal follow‐up of four candidate *N‐*glycans in seven patients showed that those *N‐*glycans were increased in patients with poor prognosis).

**Figure 5:**
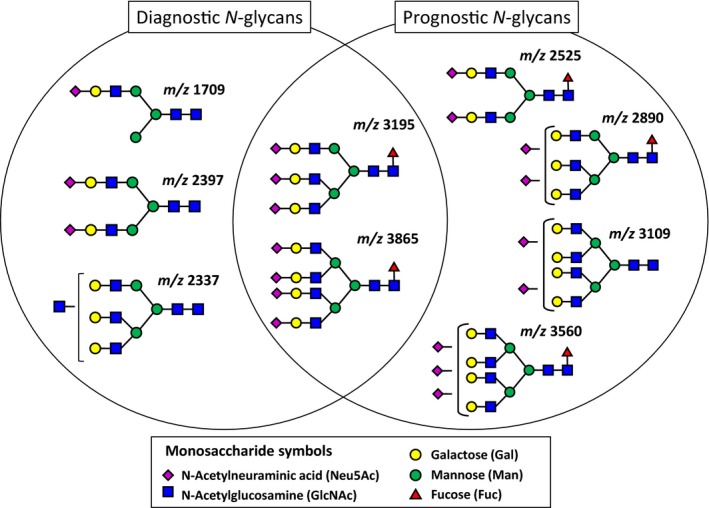
Representative diagrams of candidate *N‐*glycans in this study. Terminal sialylated bi‐antennary, tri‐antennary, and tetra‐antennary complex‐type *N‐*glycans were selected as a GCT‐related *N‐*glycans.

The predictive value of the prognostic *N‐*glycan score was significant with an AUC value of 0.89 (Fig. 4A). A high value prognostic *N‐*glycan score is significantly associated with poor prognosis (*P *=* *0.0012) (Fig. 4B). Longitudinal follow‐up of the candidate four *N‐*glycans in seven patients showed that those *N‐*glycans were increased in patients with poor prognosis (**, Figs. 4B, C, D, and E). Representative structures of the candidate *N‐*glycans identified in this study are shown in Fig. 5. Terminal sialylated bi‐antennary, tri‐antennary, and tetra‐antennary complex‐type *N‐*glycans were selected as GCT‐related *N‐*glycans.

**Figure 6:**
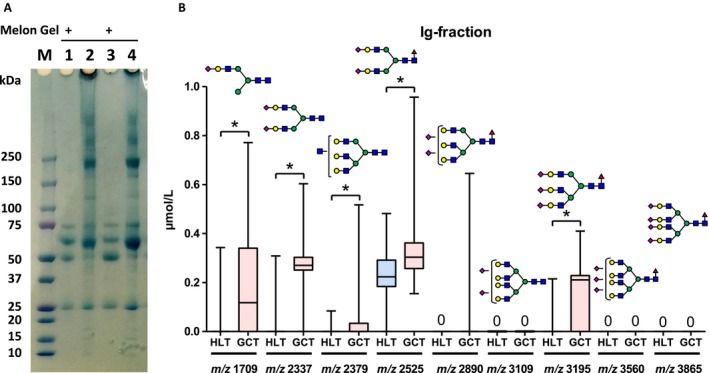
Analysis of candidate *N‐*glycans from Ig‐fractions. To address a potential carrier protein in serum, whole serum was subjected to Ig purification assay by Melon Gel. (A) Non‐Ig proteins were eliminated, and Igs were enriched by Melon Gel purification (Lane 1 and 3) compared with whole serum (lane 2 and 4). (B) Next, Ig‐fractions were subjected to *N‐*glycan analysis (B).

To address the potential carrier protein in serum, whole serum was subjected to an Ig purification assay by Melon Gel. SDS‐PAGE gel assay and Coomassie Brilliant Blue staining confirmed the amount of protein in serum. Representative Ig‐fractions are shown in Fig. 6A. Non‐Ig proteins were eliminated, and Igs were enriched by Melon Gel purification (Lane 1 and 3) compared with whole serum (lane 2 and 4) (Fig. 6A). Whole serum and the Ig‐fraction were subjected to *N‐*glycan analysis. *N‐*glycans in the Ig‐fractions were significantly decreased as compared with whole sera (Fig. 6B, S1). Of note, highly branched complex‐typed *N‐*glycans (*m/z* 3109, 3560, 3865) were below the measurement limit in the Ig‐fraction. However, concentrations of bi‐antennary (*m/z* 1591, 1607, 1753, 1915, 2058) and bisecting (*m/z* 1794) *N‐*glycans, which were not selected as GCT‐related *N‐*glycans, were not changed in Ig‐fractions (Fig. S2).

## Discussion

Quantitative *N‐*glycan analysis is expected to be a useful tool in the diagnosis of diseases 5. However, the crucial bottleneck for structural and functional glycan analysis is a time‐consuming multistep process to purify small amounts of glycans from highly complicated mixtures. Therefore, we used a recently established high‐throughput, quantitative *N‐*glycomics technology. The usefulness of this method of *N‐*glycan analysis for renal‐cell carcinoma 11, prostate cancer 10, 15, pancreatic cancer 16, bladder cancer 14, and hepatocellular carcinoma 17 has been reported. However, no study investigated the use of serum *N‐*glycans as a diagnostic and prognostic marker for GCT. To the best of our knowledge, this is the first report to demonstrate the clinical significance of serum *N‐*glycan profiling as a biomarker for GCT.

In this study, we identified that a combination of GCT‐related *N‐*glycans (*N‐*glycan score) has the potential to be a novel serum biomarker for GCT detection and prognosis. Our results also suggested that it was able to detect conventional tumor marker‐negative patients with a high rate (83%), suggesting a potential of usefulness for follow‐up when conventional tumor markers are negative, or for evaluation in retroperitoneal residual lesion after chemotherapy. On the other hand, histological subtypes of GCT were not related to *N‐*glycan score, suggesting that these *N‐*glycans are not related to tumor‐producing proteins, such as HCG and/or AFP. Currently, the usefulness of serum *N‐*glycans for monitoring tumor relapse in GCT patients is under investigation. Our next study will address these issues.

Our data showed that tri‐antennary and tetra‐antennary complex glycans with terminal sialylation were increased in GCT patients, which was similar to castration‐resistant prostate cancer. Fucosylated, highly branched, and sialylated *N‐*glycans are closely related to renal‐cell carcinoma in cancer detection and prognosis. It remains unclear why modification of *N‐*glycans occurs, and what type of carrier proteins are involved in *N‐*glycan modifications in sera. Because immunoglobulins are major *N‐*glycosylated proteins in serum 4 and glycosylation of immunoglobulin has critical role in developing diseases 18, 19, we hypothesized that glycosylation of immunoglobulin may play a key role for anticancer activity. However, our data showed that carrier *N‐*glycan concentrations were below the limit of measurement in Ig‐fractions in sera. Concentrations of candidate *N‐*glycans in Ig‐fractions were significantly decreased compared to whole sera (Fig. S1), whereas the amounts of immunoglobulin were not decreased in SDS‐PAGE gel staining (Fig. 6A). In contrast, concentrations of bi‐antennary *N‐*glycans (*m/z* 1591, 1607, 1753, 1794, 1915, 2058) did not differ (Fig. S2). This result indicates that GCT‐related *N‐*glycans are not strongly associated with immunoglobulins.

Another possibility is free serum *N‐*glycans. A recent study demonstrated that levels of di‐sialylated‐free *N‐*glycans in sera were increased in patients with hepatocellular carcinoma (*n* = 10), as compared with normal controls (*n* = 10) 20. From this observation, the authors hypothesized that the origin of the sialylated‐free *N‐*glycans might be the liver, because serum glycoproteins are produced by the liver. Their results suggested the possibility that the liver delivered free *N‐*glycans may play a role in sera. However, these results showed 100‐fold lower amounts of di‐sialylated‐free *N‐*glycans than we observed. This suggests that our high‐throughput *N‐*glycan analysis detects not only serum‐free *N‐*glycans, but also glycoproteins.

An additional possible carrier protein is AGP. AGP is a major plasma glycoprotein with a molecular weight of 41–43 kD and highly branched *N‐*linked glycans, which is synthesized in and secreted from the liver into plasma 21. AGP has been investigated as an acute‐phase serum glycoprotein that possesses 5*N‐*linked complex‐type heteroglycan side chains, which may be present as bi‐antennary, tri‐antennary, and tetra‐antennary structures. Additionally, it been studied in association with inflammation, autoimmune diseases, and cancer 22. Although the origin and clinical implications of serum *N‐*glycans remains unclear, our ongoing studies address these issues and demonstrate potential clinical utility.

The limitations of this study include the relatively small sample size, retrospective nature, selection bias, and nonclinical setting. In addition, patients of testicular disease other than cancer may be better controls than healthy individuals. The usefulness in regular follow‐up for relapses is unclear. Because our understanding of serum *N‐*glycans is insufficient, further study is necessary to understand the mechanism of serum *N‐*glycan formation. In addition, a large‐scale prospective cohort validation study is necessary. Despite these limitations, the strength of this study is that it is the first report to assess the utility of serum *N‐*glycan analysis for prognosis and diagnosis in GCT patients. Using a serum‐based analysis, we were able to demonstrate an independent association between serum *N‐*glycans and GCT detection and prognosis. Our findings may identify those who are at high risk of relapse, especially in conventional tumor marker‐negative patients.

## Conclusion

Although this study is small and preliminary, our results suggest that the serum *N‐*glycan profiles acquired by glycoblotting and MALDI‐TOF mass spectrometry have potential utility as a biomarker for the presence of GCT. Future large‐scale prospective validation studies may determine the clinical significance of these carbohydrate biomarkers for GCT.

## Conflicts of Interest

The authors declare no potential conflicts of interest.

## Supporting information


**Figure S1.** Comparison of candidate *N‐*glycans from Ig‐fractions and whole sera. Whole serum and Ig‐fraction were subjected to *N‐*glycan analysis
**Figure S2.** *N‐*glycans that were not decreased in Ig‐fractions.

**Table S1**. The representative 36 types of *N*‐glycans with quantitative reproducibility among all samples.
