# Supplementary figures and images for: Clinical implications of serum N‐glycan profiling as a diagnostic and prognostic biomarker in germ‐cell tumors
Source: Cancer Med. 2017 Mar 20;6(4):739–48. doi: 10.1002/cam4.1035 (PMC5387168; doi:10.1002/cam4.1035)

Fig. S1

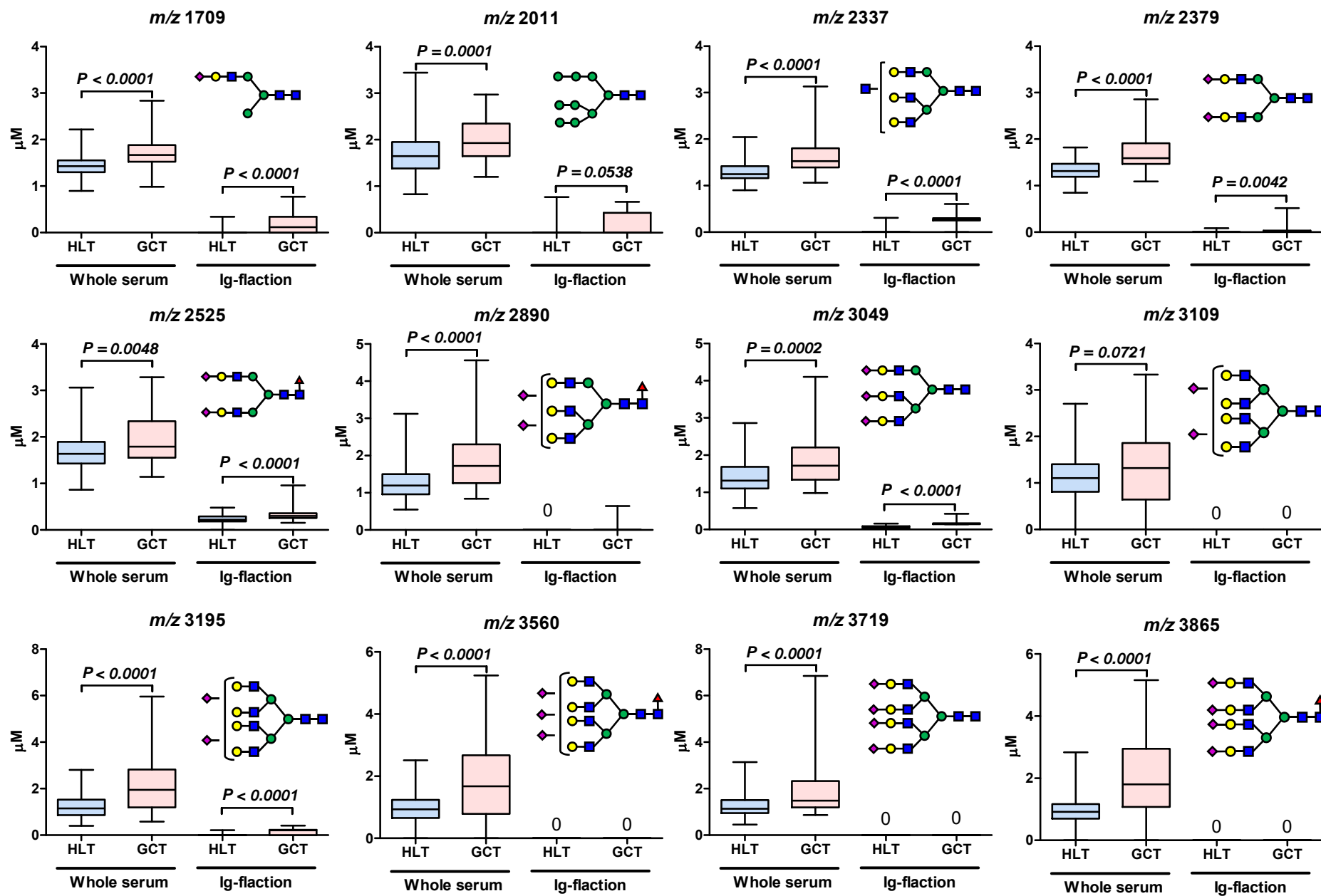

Fig. S2

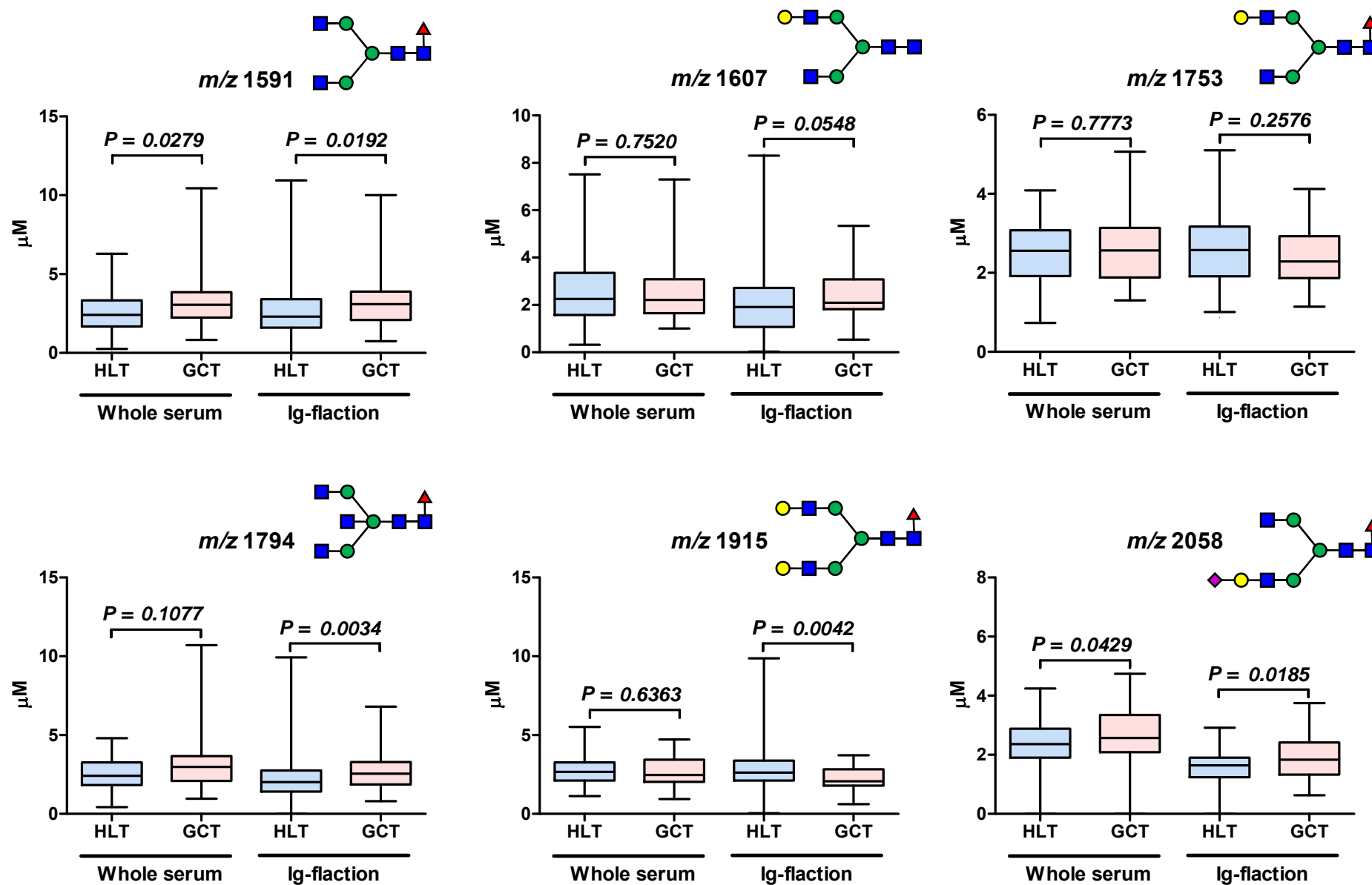

Supplement: Supplementary file 1 — Figure S1. Comparison of candidate N‐glycans from Ig‐fractions and whole sera. Whole serum and Ig‐fraction were subjected to N‐glycan analysis Figure S2. N‐glycans that were not decreased in Ig‐fractions. [file CAM4-6-739-s001.pdf]
