# Supplementary material for: Clinical implications of serum N‐glycan profiling as a diagnostic and prognostic biomarker in germ‐cell tumors
Source: Cancer Med. 2017 Mar 20;6(4):739–48. doi: 10.1002/cam4.1035 (PMC5387168; doi:10.1002/cam4.1035)
Supplement: Supplementary file 2 — Table S1. The representative 36 types of N‐glycans with quantitative reproducibility among all samples. [file CAM4-6-739-s002.doc]

**Table S1**: The representative 36 types of *N*-glycans with quantitative reproducibility among all samples. The *N*-glycan of *m/z* 2176 is the internal standard, disialo-galactosylated biantennary *N*-glycan, that contains amidated sialic acids (A2 amide glycans). Compositional annotations and putative structures are shown as abbreviations. Hex: hexose; HexNAc: *N*-acetylhexosamine; dHex: deoxyhexose.

| Peak No. | *m/z* | Composition |
| --- | --- | --- |
| 1 | 1362.5 | (Hex)2 + (Man)3(GlcNAc)2 |
| 2 | 1524.5 | (Hex)3 + (Man)3(GlcNAc)2 |
| 3 | 1565.6 | (Hex)5(HexNAc)3 |
| 4 | 1590.6 | (HexNAc)2(dHex)1 + (Man)3(GlcNAc)2 |
| 5 | 1606.6 | (Hex)1(HexNAc)2 + (Man)3(GlcNAc)2 |
| 6 | 1647.6 | (HexNAc)3 + (Man)3(GlcNAc)2 |
| 7 | 1686.6 | (Hex)4 + (Man)3(GlcNAc)2 |
| 8 | 1708.6 | (Hex)1(HexNAc)1(NeuAc)1 + (Man)3(GlcNAc)2 |
| 9 | 1752.6 | (Hex)1(HexNAc)2(dHex)1 + (Man)3(GlcNAc)2 |
| 10 | 1768.6 | (Hex)2(HexNAc)2 + (Man)3(GlcNAc)2 |
| 11 | 1793.7 | (HexNAc)3(dHex)1 + (Man)3(GlcNAc)2 |
| 12 | 1809.7 | (Hex)1(HexNAc)3 + (Man)3(GlcNAc)2 |
| 13 | 1848.6 | (Hex)5 + (Man)3(GlcNAc)2 |
| 14 | 1854.7 | (Hex)1(HexNAc)1(dHex)1(NeuAc)1 + (Man)3(GlcNAc)2 |
| 15 | 1870.7 | (Hex)2(HexNAc)1(NeuAc)1 + (Man)3(GlcNAc)2 |
| 16 | 1914.7 | (Hex)2(HexNAc)2(dHex)1 + (Man)3(GlcNAc)2 |
| 17 | 1955.7 | (Hex)1(HexNAc)3(dHex)1 + (Man)3(GlcNAc)2 |
| 18 | 2010.7 | (Hex)6 + (Man)3(GlcNAc)2 |
| 19 | 2032.7 | (Hex)3(HexNac)1(NeuAc)1 + (Man)3(GlcNAc)2 |
| 20 | 2057.8 | (Hex)1(HexNAc)2(dHex)1(NeuAc)1 + (Man)3(GlcNAc)2 |
| 21 | 2073.8 | (Hex)2(HexNAc)2(NeuAc)1+ (Man)3(GlcNAc)2 |
|  | 2175.8 | Internal standard (BOA-labeled A2 amide) |
| 22 | 2219.8 | (Hex)2(HexNAc)2(dHex)1(NeuAc)1 + (Man)3(GlcNAc)2 |
| 23 | 2336.9 | (Hex)3(HexNAc)4 + (Man)3(GlcNAc)2 |
| 24 | 2378.9 | (Hex)2(HexNAc)2(NeuAc)2 + (Man)3(GlcNAc)2 |
| 25 | 2524.9 | (Hex)2(HexNAc)2(dHex)1(NeuAc)2 + (Man)3(GlcNAc)2 |
| 26 | 2728.0 | (Hex)2(HexNAc)3(dHex)1(NeuAc)2 + (Man)3(GlcNAc)2 |
| 27 | 2744.0 | (Hex)3(HexNAc)3(NeuAc)2 + (Man)3(GlcNAc)2 |
| 28 | 2890.1 | (Hex)3(HexNAc)3(dHex)1(NeuAc)2 + (Man)3(GlcNAc)2 |
| 29 | 3049.1 | (Hex)3(HexNAc)3(NeuAc)3 + (Man)3(GlcNAc)2 |
| 30 | 3109.1 | (Hex)4(HexNAc)4(NeuAc)2 + (Man)3(GlcNAc)2 |
| 31 | 3195.2 | (Hex)3(HexNAc)3(dHex)1(NeuAc)3 + (Man)3(GlcNAc)2 |
| 32 | 3341.2 | (Hex)3 (HexNAc)3 (Deoxyhexose)2 (NeuAc)3 + (Man)3(GlcNAc)2 |
| 33 | 3414.2 | (Hex)4(HexNAc)4(NeuAc)3 + (Man)3(GlcNAc)2 |
| 34 | 3560.3 | (Hex)4(HexNAc)4(dHex)1(NeuAc)3 + (Man)3(GlcNAc)2 |
| 35 | 3719.3 | (Hex)4(HexNAc)4(NeuAc)4 + (Man)3(GlcNAc)2 |
| 36 | 3865.4 | (Hex)4(HexNAc)4(dHex)1(NeuAc)4 + (Man)3(GlcNAc)2 |
